# The impact of gastroesophageal reflux disease on upper esophageal sphincter function: Insights from PH impedance and high‐resolution manometry

**DOI:** 10.14814/phy2.70011

**Published:** 2024-08-18

**Authors:** Blake Bentley, Fadi Chanaa, Alexa Cecil, Steven Clayton

**Affiliations:** ^1^ Department of Internal Medicine Wake Forest University School of Medicine Winston‐Salem North Carolina USA; ^2^ Section on Gastroenterology and Hepatology. Department of Internal Medicine Wake Forest University School of Medicine Winston‐Salem North Carolina USA

**Keywords:** acid exposure time, GERD, high‐resolution manometry, pH impedance testing, upper esophageal sphincter

## Abstract

Lower esophageal sphincter (LES) pathophysiology has been established in gastroesophageal reflux disease (GERD); however, less is understood regarding the role the upper esophageal sphincter (UES) plays in preventing laryngopharynphageal reflux. Sustained UES basal pressure prevents reflux into the pharynx while allowing relaxation during ingestion. We investigate whether GERD influences UES function via HRM and pH Impedance testing. A retrospective analysis of 318 patients who underwent high‐resolution manometry with trans‐nasally placed manometric catheter and 24‐h multichannel intraluminal impedance pH monitoring. One hundred and forty‐seven patients met Lyon consensus criteria for GERD based on acid exposure time >6%. The most common chief concern was heartburn or reflux, present in 59% of these patients. Upper esophageal sphincter basal and residual pressures were not significantly different between patients with GERD when compared to those without GERD, including a subanalysis of patients with extraesophageal symptoms. The LES basal and residual pressures, DCI and MNBI are statistically lower in patients with pathologic GERD. HRM and pH Impedance testing demonstrates no difference in UES basal and residual pressures based on pH diagnosis of GERD. We redemonstrate the association with hypotonic LES, diminished DCI and MNBI with GERD.

## 1 INTRODUCTION

Gastroesophageal reflux disease (GERD) is the most common disorder of the gastrointestinal tract with approximately 20% of individuals in the United States experiencing symptoms at least weekly (El‐Serag et al., 2014; Eusebi et al., 2018). There are over 100,000 hospital admissions and approximately 4% of primary care clinic visits annually due to GERD symptoms (Dent et al., 2005; Thukkani & Sonnenberg, 2010). Physiologic amounts of gastroesophageal reflux are normal with natural protective mechanisms of the esophagus preventing mucosal damage and troublesome symptoms.

Ambulatory pH testing is one such minimally invasive procedure that can confirm the diagnosis in patients with either atypical symptoms or inadequate response to medical therapy, such as proton pump inhibitors. In 1974, Johnson and Demeester originally created a composite score of ambulatory pH monitoring values for the diagnosis of GERD. The primary measurement was the probe's total acid exposure time (AET) with a pH less than 4.2%. Additional parameters included secondary measurements of total acid exposure time, number of reflux events, and length of reflux events (Johnson & Demeester, 1974). More recently, the Lyon consensus was developed to include a more stringent definition stating that AET greater than six percent is inclusive of GERD while an AET between four and six percent is indeterminate (Gyawali et al., 2024).

Esophageal manometry is a diagnostic test complementary to ambulatory pH testing as it not only directs accurate placement of pH probes used in ambulatory pH monitoring, but it also provides valuable information regarding esophageal motility associated with any episodes of reflux. This is important as the lower esophageal sphincter (LES) acts as an anti‐reflux barrier composed of tonically contracted smooth muscle, and high incidences of transient lower esophageal sphincter relaxation have been associated with GERD (Pandolfino et al., 2003). As such, many patients undergo both tests sequentially.

The development of high‐resolution manometry (HRM) has introduced more closely positioned sensors allowing for better characterization of the upper esophageal sphincter (UES) (Norton et al., 2021). The UES is composed of striated muscle that is tonically contracted during basal pressure to prevent reflux into the pharynx while relaxation or residual pressure allows for ingestion of food and venting of gas from the stomach (Bhatia & Shah, 2013). Studies have shown that retrograde flow of air causes lower esophageal sphincter relaxation leading to proximal esophageal distension and relaxation of the UES for the venting of air into the pharynx. Liquid refluxate, as in GERD, results in reflexive closure of the UES and glottis to protect from aspiration. However, data are lacking in the way reflux may affect striated versus smooth muscle tissue differently, potentially resulting in differing responses of the UES and LES, respectively.

While the physiology of LES has been well studied in patients with GERD, the role of the UES in GERD patients is not well‐established. This study analyzes symptomatic patients who have undergone HRM and pH impedance to determine if UES is tonically hypertensive as a mechanism of aspiration defense.

## 2 METHODS

A retrospective EMR query was conducted at a tertiary neurogastroenterology and motility center in to create a database of all patients who had undergone an esophagogastroduodenoscopy (EGD), pH impedance, and HRM between 09/01/2018 and 03/01/2022 were included. Institutional Review Board approval was obtained, and written consent was not required due to the deidentified nature of patient data. Patient demographics, including sex, date of birth, age at time of HRM testing, BMI, smoking history of greater than 10 pack years, alcohol use greater than seven standard drinks per week, and current opioid use were collected for each patient. EGD findings were categorized as either erosive or nonerosive.

Inclusion criteria consisted of patients at least 18 years of age and with the above testing within 6 months of each other. The range of presenting symptoms included heartburn, regurgitation, chest pain, dysphagia, odynophagia, nausea, vomiting, cough, hoarseness, and globus sensation. Patients less than 18 years old, on antisecretory agents, such as proton pump inhibitor (PPI) therapy, at time of pH impedance testing were omitted from the study. There were no exclusion criteria for pre‐existing motility disorders to provide a more representative sample of the population.

UES basal and residual pressures in patients with pathologic GERD based on distal acid exposure on pH impedance testing were compared to controls with negative testing on pH Impedance. Further subanalysis was performed by defining normal pH impedance by both distal AET < 4% and AET > 6%, according to Lyon consensus criteria, as well as combining impedance data with total reflux episodes, AET <4% + TRE < 40 and AET >6% + TRE > 80. Lyon 2.0 consensus criteria recommends utilizing AET >4% + TRE > 80 in patients with an indeterminate AET; however, we chose a more stringent value of meeting criteria based on both AET and TRE.

### 2.1 High resolution manometry

All subjects additionally underwent HRM testing and were instructed to fast 6 h prior to the procedure. The manometric catheter was placed trans‐nasally until the tip was secured in the esophagogastric junction. The patient was placed in supine position and asked to swallow 5 mL of water 10 times followed by a multiple rapid swallow sequence consisting of five swallows. The patient was then moved to an upright position for five liquid swallows of 5 mL of water followed by a rapid drink challenge of 200 mL of water through a straw. The following variables were recorded for each subject: LES basal pressure, integrated relaxation pressure (IRP), UES basal pressure, UES residual pressure, average distal contractile integral (DCI), and distal latency.

### 2.2 pH impedance testing

Each study subject previously underwent 24‐h multichannel intraluminal impedance with pH monitoring (MII‐pH). Patients were instructed to fast 6 h prior to pH probe insertion. The dual‐probe pH monitor sensors are placed trans‐nasally at 10 cm below and 5 cm above the LES as determined by manometry allowing simultaneous monitoring of both gastric acid secretion and esophageal acid exposure. Patients were then asked to keep the probe inserted throughout the day while keeping a diary of meals, symptoms, or malfunctions; however, a standard meal was not utilized, and food and drink were at the patients' discretion. The following variables were recorded for each subject: total acid exposure time (AET) (%), upright AET (%), recumbent AET (%), total reflux episodes, mean nocturnal baseline impedance (MNBI) (ohms), and longest reflux (min).

### 2.3 Statistical analysis

To assess differences between the various groups, we utilized the Mann–Whitney U test (Wilcoxon rank‐sum test) due to the non‐normal distribution of our data and the independent nature of our samples. The test compared the median scores of the two groups, ranking all values together and evaluating if ranks significantly differed between them with a significance level set at 0.05. Effect size was also determined to quantify the difference magnitude between the groups.

## 3 RESULTS

During the study period, 727 adults were identified as having underwent the appropriate testing with 409 patients excluded either due to the testing being greater than 6 months apart or being on an antisecretory agent at time of pH impendence testing. In total, 318 patients met inclusion criteria. The average age was 53 years with an average BMI of 31.2. 76.1% of patients were female. Tobacco use was present in 75 (24%) patients, alcohol use in 25 (8%), and opioid use in 16 (5%) patients. Patient demographics are presented in Table 1.

**TABLE 1:** Demographic information of the 318 patients meeting inclusion criteria.

Lyons pH criteria	AET >6% *n* = 147		AET <4% *n* = 122	AET 4%–6% *n* = 49
Female (%)	111 (76%)		26 (76%)	95 (73%)
Age (Mean)	53		54	54
BMI (Mean)	32.4		29.5	31.5
Hiatal hernia (%)	70 (48%)		54 (44%)	29 (59%)
Smoking history (%)	33 (22%)		26 (21%)	16 (33%)
Alcohol history (%)	9 (6%)		13 (11%)	3 (6%)
Opioid use (%)	5 (3%)		9 (7%)	2 (4%)

Utilizing Lyon consensus normative values of total acid exposure time <4% as normal, 4%–6% as indeterminate and >6% as abnormal, 147 patients (76% female) met criteria for GERD with an average AET of 15.3% (Standard deviation of 9.7%). Forty‐nine patients had an AET in the indeterminate range and 122 patients were in the normal range. Only 53 (16.7%) patients met Lyon consensus criteria for GERD based on total reflux episodes. The most common chief concern in patients diagnosed with GERD was heartburn or reflux in 59%, extraesophageal (i.e., cough, globus sensation, or hoarseness) in 15% and dysphagia in 13% of patients. Twenty‐seven patients had no reported symptoms during pH impedance of testing but had a history of GERD and/or hiatal hernia and were included in data analysis. The presence of hiatal hernia was similar between the group of patients with GERD and those without GERD (48% vs. 44%, respectively).

In the cohort of 147 patients meeting Lyon consensus criteria for GERD based on AET, the UES basal and residual pressures were compared with patients with normal AET of which statistical significance was not met. Further analysis based on Lyon consensus criteria on total number of reflux episodes (TRE), upright AET, recumbent AET, MNBI, and longest reflux showed no statistically significant differences in UES average basal and residual pressures, detailed in Table 2. Based on Lyon 2.0 consensus criteria that suggests combining impendence data with TRE, we used a more stringent criteria of AET >6% and >80 TRE and found no significant differences in UES average basal and residual pressures (*p* = 0.221 and 0.893, respectively), detailed in Table 3. In a subanalysis of patients whose primary or secondary complaint included extraesophageal symptoms (i.e., cough, globus sensation, or hoarseness), we found no statistically significant difference between UES average basal and residual pressures in patients with AET >6% and >80 TRE compared with AET <4% and <40 TRE (*p* = 0.648 and 0.926, respectively), detailed in Table 4. As the Lyon Conference guidelines are focused on the clinical application of HRM and pH‐impedance data, we subsequently divided the patients into the following GERD phenotypes: erosive esophagitis, nonerosive reflux disease with AET >6%, reflux hypersensitivity with AET <6%, and functional heartburn with AET <6%, as detailed in Table 5.

**TABLE 2:** Comparison of basal and residual pressures in the lower esophageal sphincter and upper esophageal sphincter based on various pH impedance variables.

Acid exposure time		AET <4%	AET >6%	*p* Value
LES	Basal (mmHg)	37.7 (SD 17.7)	33.7 (SD 15.1)	0.08
	Residual (mmHg)	15.7 (SD 9.7)	13.7 (SD 8.0)	0.10
UES	Basal (mmHg)	64 (SD 33.4)	70.1 (SD 37.8)	0.30
	Residual (mmHg)	18.1 (20.6)	20.4 (SD 26.8)	0.90
DCI (mmHg‐cm‐s)		2320.2 (SD 1994.8)	1987.4 (SD 1901.2)	0.03*

# Reflux events		# Reflux <40	# Reflux >80	
LES	Basal (mmHg)	36.76 (SD 17.5)	32.07 (SD 16.0)	0.07
	Residual (mmHg)	16.15 (SD 10.2)	12.12 (SD 7.3)	0.01*
UES	Basal (mmHg)	66.72 (SD 36.2)	72.41 (SD 36.8)	0.23
	Residual (mmHg)	17.92 (SD 19.6)	18.23 (SD 24.2)	0.61

Upright AET		AET <6.3%	AET >6.3%	
LES	Basal (mmHg)	35.06 (SD 16.4)	36.22 (SD 16.4)	0.52
	Residual (mmHg)	15.09 (SD 9.1)	14.58 (SD 8.7)	0.62
UES	Basal (mmHg)	69.56 (SD 38.9)	69.25 (SD 38.3)	0.76
	Residual (mmHg)	18.5 (SD 9.1)	19.85 (SD 9.9)	0.77

Recumbent AET		AET <1.2%	AET >1.2%	
LES	Basal (mmHg)	37.81 (SD 16.6)	34.35 (SD 16.2)	0.05*
	Residual (mmHg)	16.21 (SD 9.8)	14.05 (SD 8.2)	0.05*
UES	Basal (mmHg)	68.66 (SD 37.2)	69.84 (SD 39.5)	0.99
	Residual (mmHg)	20.54 (SD 22.7)	18.35 (SD 23.7)	0.08

MNBI		MNBI >2292	MNBI <2292	
LES	Basal (mmHg)	37.47 (SD 17.0)	34.61 (SD 16.4)	0.30
	Residual (mmHg)	15.72 (SD 8.6)	13.38 (SD 7.7)	0.07
UES	Basal (mmHg)	67.29 (SD 35.1)	71.02 (SD 39.0)	0.56
	Residual (mmHg)	24.61 (SD 24.2)	22.32 (SD 26.3)	0.15

Longest reflux		Reflux <9.2 min	Reflux >9.2 min	
LES	Basal (mmHg)	38.2 (SD 17.4)	34.2 (SD 15.6)	0.04*
	Residual (mmHg)	16.5 (SD 9.5)	14.0 (SD 8.4)	0.01*
UES	Basal (mmHg)	67.1 (SD 37.6)	70.7 (SD 39.1)	0.55
	Residual (mmHg)	19.0 (SD 21.5)	19.2 (SD 24.4)	0.99

* *p* < 0.05.

**TABLE 3:** Comparison of basal and residual pressures in the lower esophageal sphincter and upper esophageal sphincter, distal contractile integral and mean number of baseline impedance in patients with objective evidence of GERD.

		AET <4% and TRE < 40 (*n* = 71)	AET >6% and TRE >80 (*n* = 34)	*p* Value
AET	%	1.6 (SD 1.2)	15.8 (SD 9.3)	<0.001*
TRE	Total number	23.5 (SD 10.5)	117.8 (SD 40.9)	<0.001*
LES	Basal (mmHg)	37.1 (SD 19.1)	28.7 (SD 11.4)	0.027*
	Residual (mmHg)	16.7 (SD 11.1)	12.2 (SD 6.5)	0.046*
UES	Basal (mmHg)	65.4 (SD 33.9)	75.8 (SD 41.5)	0.221
	Residual (mmHg)	18.9 (SD 21.0)	19.3 (SD 27.2)	0.893
DCI	mmHg‐cm‐s	2349 (SD 2161)	1374 (SD 1021)	0.012*
MNBI	mmHg	3136 (SD 1362)	1863 (SD 1027)	<0.001*

* *p* < 0.05.

**TABLE 4:** Comparison of basal and residual pressures in the lower esophageal sphincter and upper esophageal sphincter, distal contractile integral and mean number of baseline impedance in patients with objective evidence of GERD and extraesophageal symptoms.

		AET <4% and TRE < 40 (*n* = 25)	AET >6% and TRE >80 (*n* = 17)	*p* Value
AET	%	1.9 (SD 1.3)	16.6 (SD 10.6)	<0.001*
TRE	Total number	26.7 (SD 10.5)	132.6 (SD 51.2)	<0.001*
LES	Basal (mmHg)	37.2 (SD 17.8)	27.6 (SD 12.3)	0.047*
	Residual (mmHg)	17.7 (SD 10.8)	10.3 (SD 6.6)	0.024*
UES	Basal (mmHg)	65.6 (SD 35.2)	82 (SD 47.3)	0.282
	Residual (mmHg)	16.3 (SD 16.7)	25.9 (SD 34.1)	0.356
DCI	mmHg‐cm‐s	2976 (SD 2995)	1347 (SD 1188)	0.016*
MNBI	mmHg	3470 (SD 1166)	1769 (SD 912)	0.001*

* *p* < 0.05.

**TABLE 5:** Comparison of basal and residual pressures (mmHg) in the upper esophageal sphincter and average distal contractile integral (mmHg‐cm‐s) between GERD phenotypes.

GERD phenotype	Frequency, *n* (%)	AET (SD)	UES basal pressure (SD, *p* value)	UES residual pressure (SD, *p* value)	Average distal contractile integral (SD, *p* value)
Functional heartburn	44 (14%)	2.36 (1.72)	67.22 (41.94)	14.23 (16.18)	2201.86 (1618.16)
Reflux hypersensitivity	122 (38%)	2.92 (1.57, 0.03*)	71.36 (37.93, 0.32)	22.80 (22.39, 0.02*)	2576.98 (2367.22, 0.84)
Non‐erosive reflux disease	63 (20%)	15.09 (9.62, <0.01*)	68.31 (34.73, 0.46)	18.67 (24.67, 0.93)	2126.29 (2000.33, 0.14)
Erosive esophagitis	89 (28%)	10.62 (10.08, <0.01*)	72.82 (43.34, 0.46)	25.22 (30.65, 0.15)	1482.70 (1034.41, 0.01*)

* *p* = <0.05.

A secondary aim was to evaluate the LES average basal and residual pressures of this cohort of 147 patients meeting Lyon consensus criteria for GERD based on AET, TRE, upright AET, recumbent AET, MNBI and longest reflux, as detailed in Table 2. We demonstrated statistically different DCI based on AET (2320.2 vs. 1987 mmHg‐cm‐s, *p* = 0.03), LES residual pressure (16.1 vs. 12.1 mmHg, *p* = 0.01) based on TRE, basal and residual pressures based on upright AET as well as longest reflux. Using the combined criteria of AET >6% and TRE >80, both the basal and residual LES pressures were statistically different from AET < % and TRE <40, as detailed in Table 3.

## 4 DISCUSSION

**FIGURE 1:**
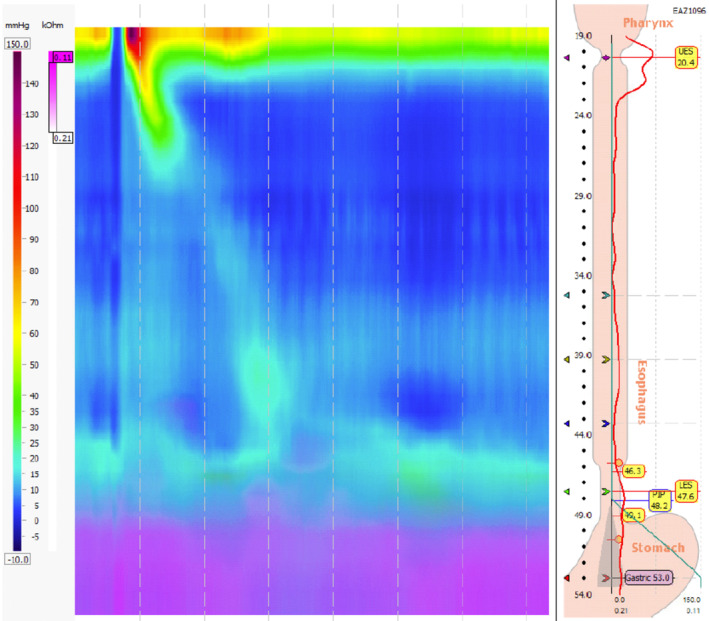
High‐resolution manometry demonstrating a hypotensive relaxing LES with intact but inadequate peristalsis and a mean DCI <200 mmHg‐cm‐s (450–5000), consistent with ineffective esophageal motility.

The primary esophageal mechanisms by which the esophagus attempts to limit reflux of gastric contents, including lower esophageal sphincter, buffer effect of saliva, epithelial resistance factors, esophageal peristalsis and, lastly, upper esophageal sphincter (Brown & Shermetaro, 2023). Hypotensive basal lower esophageal sphincter pressure has previously been shown as one of the best predictors of pathological acid reflux in GERD (Jain et al., 2018). The symptoms of GERD can range from mild to severe. We assessed UES and LES basal and residual pressures through HRM and pH Impedance testing related to AET, reflux events, and time of reflux. Our study found that there is no statistical difference in UES pressures in patients with or without GERD (Figure 1).

Numerous studies evaluating the upper esophageal sphincter have attempted to delineate the relationship between UES pressure in GERD with differing results. The UES reflex arc has been studied utilizing multiple modalities throughout the literature. This was further delineated by Pandolfino et al. into esophago‐upper sphincter contraction and relaxation reflex with HRM (Pandolfino et al., 2003). The UES contraction reflex is thought to be mediated by the muscular layer of the esophagus via slow adapting mechanoreceptors while the UES contraction reflex is a function of mucosal rapid adapting receptors (Szczesniak et al., 2008). Conceptually, one would theorize that the UES is reflexively hypertensive to protect against aspiration, and with distal acid exposure, this mechanism is initiated.

Hunt et al. demonstrate both cricopharyngeal sphincter pressure and UES tone increases with distal acid exposure utilizing classic manometry (Hunt et al., 1970). Additionally, Kuribayashi et al found UES tone to be increased with obstructive sleep apnea, linked frequently with GERD and erosive esophagitis, and tone significantly higher (*p* < 0.01) at the end of OSA events compared to the beginning (Kuribayashi et al., 2010). Both studies included small cohorts of patients.

Several more recent studies have shown patients with GERD have hypotonic UES pressure when compared with healthy subjects. Passaretti et al studied 42 patients and found the median UES resting pressure and median proximal contractile integral were lower in pathological oropharyngeal acid exposure when compared with a group with normal acid exposure (Passaretti et al., 2016). Additionally, Nadaleto et al compared 44 patients under evaluation with GERD with 40 healthy volunteers and found that in patients with GERD, the manometric profile is characterized by a short and hypotonic UES that is more pronounced in patients with extraesophageal symptoms (Nadaleto et al., 2017). Conceptually, this would seem logical as a shorter and hypotonic UES would allow for increased laryngopharyngeal reflux; however, when evaluating the Nadaleto study, the difference in patient populations (average age of 61 years in GERD group versus 27 years in health controls) (Nadaleto et al., 2017) raises the question of whether this difference is a normal result of aging and whether it is truly reflective of a pathologic difference. We find that in patients with extraesophageal symptoms as their primary or secondary complaint (i.e., cough, globus sensation, or hoarseness) and objective criteria of GERD with AET >6% and TRE >80, there is no difference in the UES basal (82.0 vs. 65.6 mmHg, *p* = 0.282) or UES residual (25.9 vs. 16.3 mmHg, *p* = 0.356) pressures.

Furthermore, we stratified the patients into four GERD phenotypes, finding that the UES residual pressure is significantly different between nonerosive reflux disease and functional heartburn (22.80 vs. 14.23 mmHg, *p* = 0.02) as well as the average DCI between erosive esophagitis and functional heartburn (2201.86 vs. 1482.70 mmHg‐cm‐s, *p* = 0.01).

Our study reconfirms that in patients with GERD, the LES basal and residual pressures in the more stringent group comparing AET >6% and TRE >80 with AET <4% and TRE < 40 redemonstrate these findings of significantly lower LES basal (28.7 vs. 37.1, *p* = 0.027), residual (12.2 vs. 16.7, *p* = 0.046), DCI (1374 vs. 2349 mmHg‐cm‐s, *p* = 0.012), and MNBI (1863 vs. 3136 mmHg, *p* = < 0.001), Table 3. Both primary and secondary esophageal peristalsis are essential mechanisms of esophageal clearance of gastric fluid with decreased acid clearance and duration of AET highly related to GERD (Vaezi & Richter, 1996). DCI is a composite measure that integrates the amplitude, duration, and length of the esophageal contraction in the distal lower esophagus, representing the vigor of esophageal contractions. Patients with ineffective esophageal motility have weak esophageal contraction which can impair the esophagus' ability to clear acid, potentially worsening pathologic reflux. Our findings build upon prior studies that have suggested DCI, or the similar esophagogastric junction‐contractile integral, may be clinically effective methods of predicting abnormal AET and better surgical candidates (Gor et al., 2016; Ham et al., 2017; Rengarajan & Gyawali, 2020).

In one of the largest studies utilizing pH Impedance and HRM to assess the UES, we evaluated 318 patients with symptomatic GERD with 147 patients meeting Lyon consensus criteria. Our study demonstrated no statistical difference of the upper esophageal sphincter basal and residual pressures between patients with or without GERD when assessing AET, number of reflux events, upright AET, recumbent AET, duration of longest reflux or NMBI.

Our study has a few limitations that must be considered including potential selection bias as this was a retrospective review. As is true in prior studies evaluating the UES, the overall smaller sample size limits statistical power. Finally, Karjilas has previously shown that various substances, such as hormones, neural agents, medications, and foods can either increase or decrease the lower esophageal sphincter pressure, which raises the question of whether similar effects could be seen in the UES (Kahrilas, 1998), potentially confounding results if not properly controlled. Further investigation in the effects of acid reducing medications as well as high‐risk comorbidities, such as achalasia, gastroparesis, and hiatal hernias, can further evaluate the role of UES in GERD.

In conclusion, utilizing high‐resolution manometry and pH Impedance testing, we demonstrate there is no difference in UES basal and residual pressures based on the presence of GERD in one of the largest studies to date. Additionally, we redemonstrate the role hypotonic LES and diminished DCI have in patients with GERD.

## AUTHOR CONTRIBUTIONS

Blake Bentley was involved in data collection, statistical analysis, draft, and review of the manuscript. Fadi Chanaa was involved in data collection and draft of the manuscript. Alexa Cecil was involved in draft and review of the manuscript. Steven Clayton was involved in the development and design of the research study, editing, and approval of the final manuscript.

## FUNDING INFORMATION

None.

## ETHICS STATEMENT

The protocol for this retrospective cohort study has been approved by the Wake Forest University School of Medicine Institutional Review Board (#IRB00073190).

## Data Availability

The data that support the findings of this study are available on request from the corresponding author.
